# Resolution of Lancisi’s sign following bicaval TricValve implantation for torrential tricuspid regurgitation: a case report defining a novel bedside marker of haemodynamic recovery

**DOI:** 10.1093/ehjcr/ytag735

**Published:** 2026-10-17

**Authors:** Mohamed Asfour, Huseyin Bozbas

**Affiliations:** Department of Cardiology, TOBB University of Economics and Technology Faculty of Medicine, Yaşam Cad. No: 5, Söğütözü, Ankara 06560, Türkiye; Department of Cardiology, TOBB University of Economics and Technology Faculty of Medicine, Yaşam Cad. No: 5, Söğütözü, Ankara 06560, Türkiye

**Keywords:** Tricuspid regurgitation, Lancisi's sign, TricValve, Caval valve implantation, Case report

## Abstract

**Background:**

Caval valve implantation (CAVI) using the TricValve system is an emerging treatment option for severe tricuspid regurgitation (TR) in patients who are unsuitable for surgery or orthotopic transcatheter intervention. Lancisi's sign is a classical manifestation of severe TR; however, its resolution after CAVI has not been described.

**Case summary:**

An 85-year-old woman with recurrent right heart failure and torrential TR presented with prominent bilateral Lancisi's sign. Echocardiography revealed severe annular dilatation (48 mm), right ventricular dysfunction (TAPSE, 13 mm), and a left ventricular ejection fraction of 40%. Owing to prohibitive surgical risk (EuroSCORE II 18.4%; TRI SCORE 14), she underwent successful implantation of 29-mm superior vena cava and 35-mm inferior vena cava TricValve prostheses. At 3 months, Lancisi's sign had completely resolved, NYHA functional class improved from III-IV to I-II, NT-proBNP decreased from 25 000 to 2 000 pg/mL, TAPSE increased from 13 to 20 mm, and no rehospitalization or device-related adverse event occurred. We report a structured single-patient case using clinical examination, echocardiography, invasive hemodynamic assessment, laboratory markers, and 3-month follow-up after bicaval TricValve implantation.

**Discussion:**

Bicaval TricValve implantation resulted in marked symptomatic, biochemical, echocardiographic, and bedside clinical improvements. The resolution of Lancisi's sign may represent a simple marker of hemodynamic recovery after CAVI.

Learning points

- Severe tricuspid regurgitation can produce prominent systolic jugular venous pulsations, known as Lancisi’s sign.
- TricValve implantation can reduce systemic venous congestion in high-risk patients unsuitable for surgery or orthotopic transcatheter intervention.
- Resolution of Lancisi’s sign may provide a simple bedside marker of reduced systemic venous reflux after caval valve implantation.

## Introduction

Severe tricuspid regurgitation (TR) is increasingly recognized as an important determinant of morbidity and mortality. Chronic severe TR causes right ventricular volume overload, systemic venous congestion, recurrent hospitalization, hepatic and renal dysfunction, and impaired quality of life. Despite this burden, isolated tricuspid valve surgery is frequently avoided because affected patients are often elderly and frail and have multiple comorbidities associated with prohibitive operative risks.^1,2^

Transcatheter therapies have expanded the treatment options for this high-risk population. Caval valve implantation (CAVI) is a heterotopic approach in which bioprosthetic valves are implanted within the superior vena cava (SVC) and inferior vena cava (IVC) to prevent systolic backflow into the systemic venous circulation. The TricValve system has demonstrated favourable clinical and haemodynamic outcomes in the TRICUS EURO study and subsequent multicentre registries.^3–8^

Lancisi’s sign, originally described by Giovanni Maria Lancisi and more recently reviewed in modern clinical literature, refers to giant systolic jugular venous pulsations caused by the retrograde transmission of right atrial V waves into the jugular venous system.^9,10^ Although this sign is a well-established clinical manifestation of severe TR, its behaviour after CAVI has not previously been reported. We present a case of successful bicaval TricValve implantation in an elderly patient with torrential TR, highlighting the complete resolution of Lancisi’s sign as a novel bedside indicator of haemodynamic recovery.

## Summary figure

**Figure:**
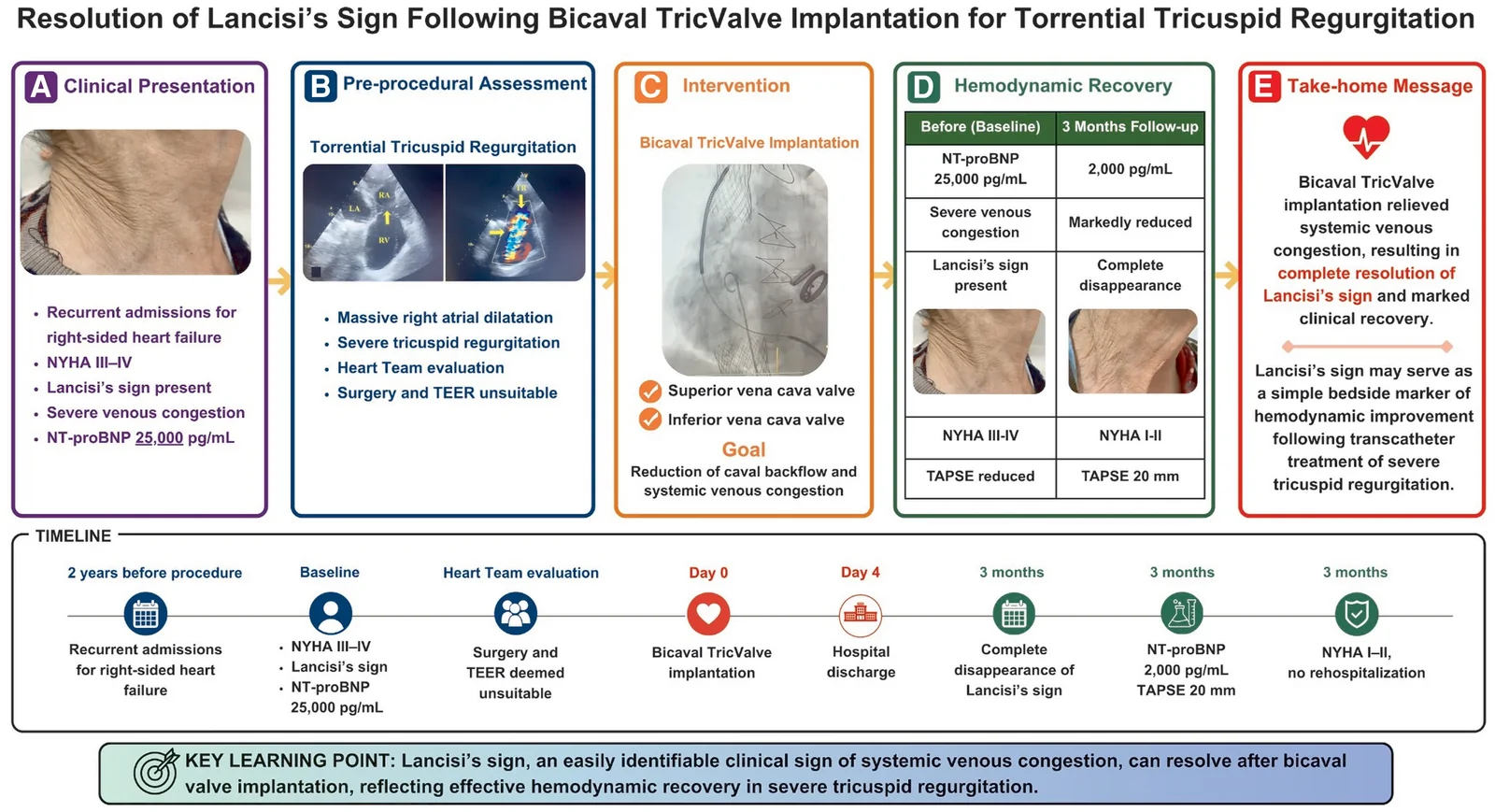
Graphical summary of the clinical presentation, treatment, and outcome of an elderly patient with torrential tricuspid regurgitation undergoing bicaval TricValve implantation. (*A*) Clinical presentation showing prominent Lancisi’s sign associated with advanced right-sided heart failure, severe systemic venous congestion, NYHA Class III–IV symptoms, and elevated NT-proBNP (25 000 pg/mL). (*B*) Pre-procedural assessment demonstrating torrential tricuspid regurgitation with marked right atrial dilatation; Heart Team evaluation deemed surgical intervention and transcatheter edge-to-edge repair unsuitable. (*C*) Successful bicaval implantation of the TricValve system in the superior and IVC to reduce systolic caval backflow and systemic venous congestion. (*D*) Three-month follow-up demonstrating marked haemodynamic and clinical improvement, including complete resolution of Lancisi’s sign, reduction in NT-proBNP to 2000 pg/mL, improvement in TAPSE from 13 to 20 mm, improvement in NYHA functional class from III–IV to I–II, and absence of rehospitalization. (*E*) Take-home message highlighting that bicaval TricValve implantation effectively relieved systemic venous congestion and that disappearance of Lancisi’s sign may serve as a simple bedside marker of haemodynamic recovery following transcatheter treatment of severe tricuspid regurgitation.

## Case presentation

An 85-year-old woman was referred to our hospital with worsening right-sided heart failure. Her medical history included rheumatic heart disease treated with surgical mitral and aortic valve replacement 20 years earlier. At the time of the initial valve surgery, mild to moderate TR was present; however, concomitant tricuspid valve intervention was not deemed necessary. Her medical history was also notable for permanent pacemaker implantation for atrial fibrillation with a slow ventricular response, chronic kidney disease Stage 3b, hypertension, and diabetes mellitus.

The patient remained clinically stable for many years following surgery. However, ∼2 years before the current presentation, she began to develop signs and symptoms of right-sided heart failure associated with worsening TR. Despite optimized medical therapy, including high-dose loop diuretics, her clinical condition gradually deteriorated, with recurrent hospitalizations for decompensated right heart failure and increasingly severe systemic venous congestion over the preceding 2 years.

At baseline, the blood pressure was 110/70 mmHg, and the heart rate was 72 b.p.m. Physical examination revealed elevated jugular venous pressure (15 cmH_2_O), prominent bilateral systolic jugular venous pulsations consistent with Lancisi’s sign (see Supplementary material online, *Video S1A*), a grade 2/6 holosystolic murmur that increased with inspiration (Carvallo’s sign), hepatomegaly with systolic hepatic pulsation, ascites, and bilateral pitting oedema. The functional status was New York Heart Association (NYHA) Class III–IV.

Laboratory testing revealed an N-terminal pro-B-type natriuretic peptide (NT-proBNP) level of 25 000 pg/mL and a serum creatinine level of 1.6 mg/dL. Transthoracic echocardiography revealed severe tricuspid annular dilatation (48 mm), marked leaflet malcoaptation, torrential central TR, severe right atrial and right ventricular enlargement, a tricuspid annular plane systolic excursion (TAPSE) of 13 mm, a left ventricular ejection fraction of 40%, and an estimated systolic pulmonary artery pressure of 45 mmHg (*Figure 1*). The surgical risk was prohibitive, with a EuroSCORE II of 18.4% and a TRI-SCORE of 14. The TRIVALVE Score was 3.5, indicating an increased risk of mortality or rehospitalization at 12 months.^11^ After multidisciplinary Heart Team review, surgery and transcatheter edge-to-edge repair were considered unsuitable because of the prohibitive procedural risk and unfavourable anatomy.

**Figure 1:**
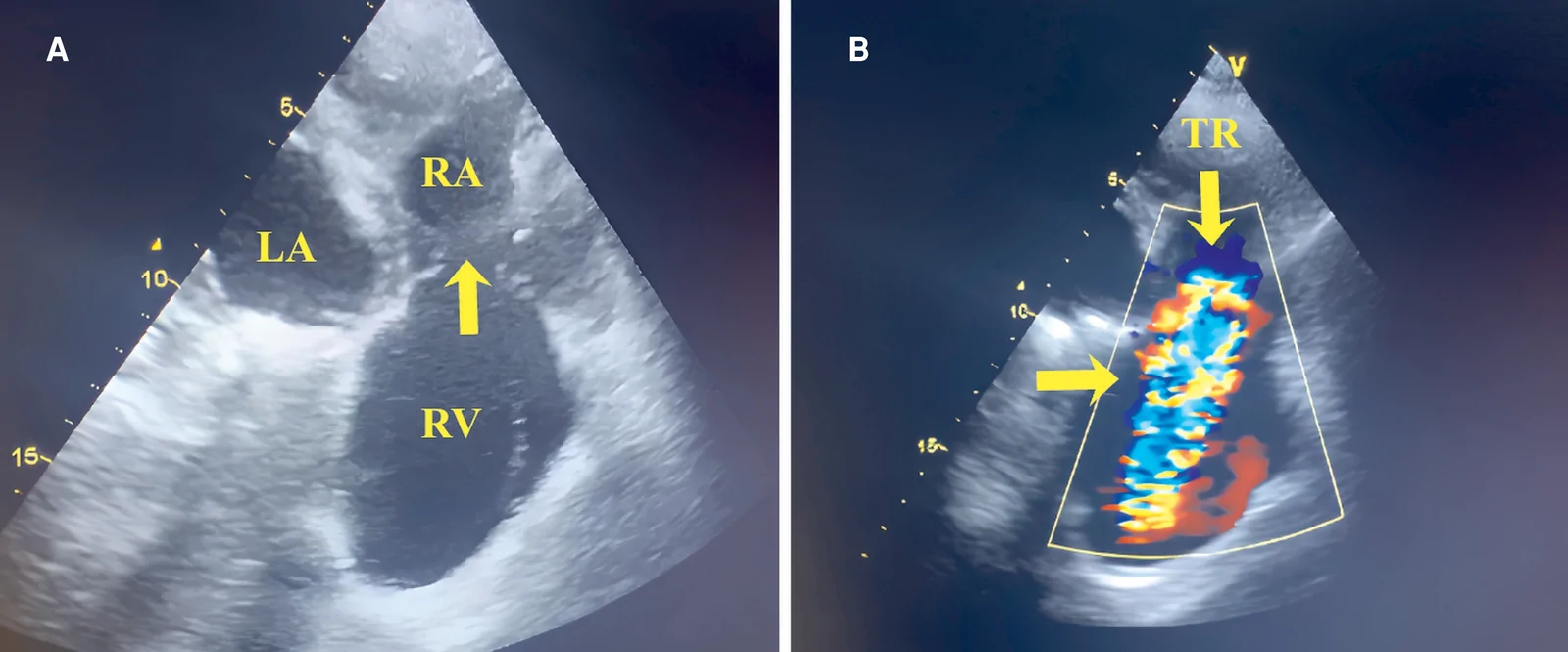
Baseline transthoracic echocardiography. (*A*) Apical four-chamber view demonstrating severe right atrial enlargement and marked tricuspid annular dilatation (48 mm) with leaflet malcoaptation (arrow). (*B*) Colour Doppler imaging demonstrating a broad tricuspid regurgitation jet occupying more than 50% of the right atrial area, consistent with torrential tricuspid regurgitation (arrow). Abbreviations: LV, left ventricle; RV, right ventricle; RA, right atrium; TR, tricuspid regurgitation.

Pre-procedural computed tomography (CT) was performed for anatomical assessment and prosthesis sizing, with measurements obtained during the diastolic phase at multiple levels of the SVC and IVC. The SVC dimensions ranged from 2.3 × 2.3 cm at the confluence to 3.9 × 4.7 cm at the SVC–right atrial junction, while the IVC measured 3.0 × 4.0 cm at the IVC–right atrial junction and 3.9 × 4.0 cm at the level of the hepatic vein confluence. Based on these CT-derived measurements and the caval anatomy, 29-mm SVC and 35-mm IVC TricValve bioprostheses were selected.

The procedure was performed under conscious sedation and transoesophageal echocardiographic guidance via right femoral venous access. A 29-mm TricValve bioprosthesis was implanted at the SVC–right atrial junction, followed by a 35-mm TricValve bioprosthesis at the IVC–right atrial junction immediately above the hepatic veins. Completion angiography confirmed optimal valve positioning without migration, obstruction, or paravalvular leakage. Invasive haemodynamic assessment showed a reduction in the right atrial V-wave amplitude from 28 to 16 mmHg after the procedure. The total procedural duration was 75 min, fluoroscopy time was 18 min, and contrast volume was 80 mL. No procedural complications occurred, and the patient was discharged on the fourth postoperative day.

At 3-month follow-up, the patient demonstrated substantial clinical, biochemical, and echocardiographic improvements. Lancisi’s sign had completely disappeared (see Supplementary material online, *Video S1B*), peripheral oedema and ascites resolved, exercise tolerance improved, and NYHA functional class improved from III–IV to I–II.

Echocardiography revealed a stable prosthetic valve position and competent caval valve function without pathological systemic or venous reflux. The mean transvalvular gradient was <3 mmHg. TAPSE improved from 13 to 20 mm, exceeding the commonly used threshold of 17 mm for preserving right ventricular systolic function.^12^

The laboratory findings also improved. The NT-proBNP level decreased from 25 000 to 2000 pg/mL, serum creatinine level improved from 1.6 to 1.3 mg/dL, and the bilirubin level normalized. The daily furosemide dose was reduced from 80 to 20 mg daily. No rehospitalization or device-related adverse events occurred during follow-up. *Figure 2* illustrates the magnitude of the improvement.

**Figure 2:**
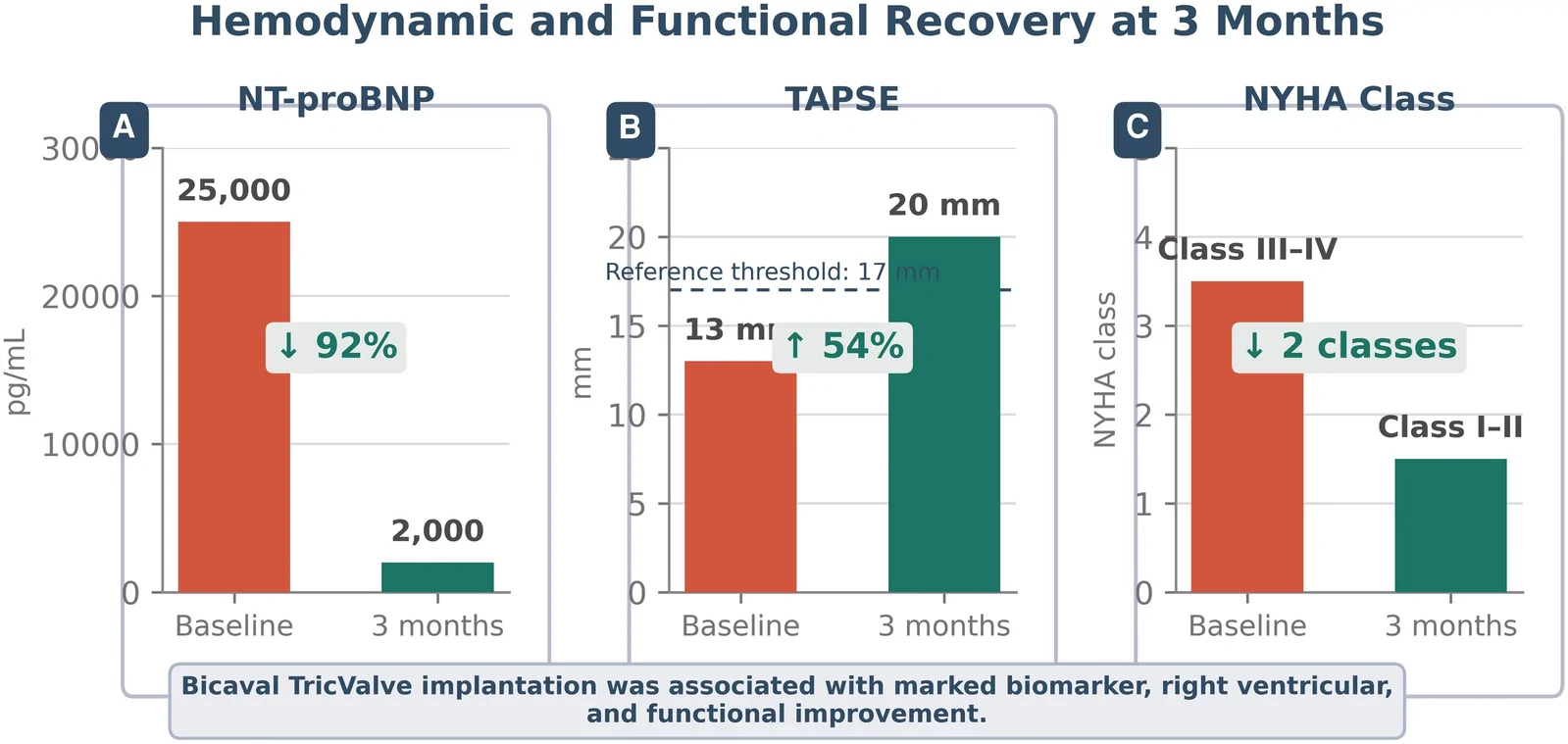
Clinical and echocardiographic recovery after bicaval TricValve implantation. (*A*) NT-proBNP decreased from 25 000 pg/mL to 2000 pg/mL (92% reduction). (*B*) TAPSE improved from 13 to 20 mm, exceeding the normal threshold of 17 mm. (*C*) Functional status improved from NYHA Class III–IV to NYHA Class I–II at the 3-month follow-up.

## Discussion

This case illustrates both the clinical efficacy and mechanistic rationale of bicaval TricValve implantation in an elderly patient with torrential TR who was unsuitable for surgery. The most novel observation is the complete disappearance of Lancisi’s sign after CAVI, occurring in parallel with improvements in symptoms, biomarkers, right ventricular function, and end-organ congestion.

Lancisi’s sign reflects the transmission of large right atrial V waves into the SVC and jugular venous system during systole.^9,10^ By preventing retrograde systolic flow into both caval veins, the TricValve system interrupts the mechanism responsible for visible venous pulsations.^3–8^ The disappearance of Lancisi’s sign in this patient is therefore mechanistically plausible and provides a direct bedside correlate of reduced systemic venous reflux.

The objective improvements observed in this patient support a true haemodynamic benefit rather than isolated symptomatic relief. NT-proBNP decreased by ∼92%, renal function improved, bilirubin levels normalized, TAPSE increased from 13 to 20 mm, and diuretic requirement decreased substantially. These changes are consistent with prior TricValve studies and registries reporting improvements in symptoms, congestion markers, functional status, and haemodynamic parameters after bicaval-valve implantation.^3–8^ Recent systematic reviews have also supported transcatheter approaches as meaningful therapeutic options for advanced TR among high-risk patients.^13^

This case also illustrates the continued relevance of physical examination in the era of structural heart disease intervention. Advanced imaging, invasive haemodynamics, and biomarkers remain essential; however, classical findings such as jugular venous distension, hepatic pulsatility, peripheral oedema, and Lancisi’s sign can provide rapid and inexpensive information on systemic venous congestion. The visible resolution of Lancisi’s sign after a contemporary transcatheter procedure highlights the complementary value of bedside assessment and advanced structural therapy.

The main limitation of this study is that it is a single-patient observation with a 3-month follow-up. Cardiac magnetic resonance imaging was not performed, and long-term clinical outcomes are unknown. Larger studies are needed to determine whether the disappearance of Lancisi’s sign consistently predicts favourable outcomes after CAVI and whether it can be incorporated as a practical clinical endpoint.

## Conclusion

Bicaval TricValve implantation achieved marked symptomatic, biochemical, echocardiographic, and bedside clinical improvement in an elderly patient with torrential TR who was unsuitable for conventional intervention. The complete disappearance of Lancisi’s sign occurred in parallel with a reduction in NT-proBNP levels, improvement in TAPSE, and resolution of systemic venous congestion. The resolution of Lancisi's sign may serve as a simple, inexpensive bedside marker of haemodynamic recovery following CAVI.

## Lead author biography


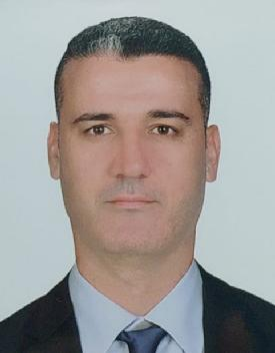


Dr Mohamed Asfour, MD, is a cardiology physician at the Department of Cardiology, TOBB University of Economics and Technology Faculty of Medicine, Ankara, Türkiye. His clinical and academic interests focus on structural heart disease, valvular interventions, heart failure, cardiovascular imaging, and transcatheter therapies. Dr Asfour has contributed to several clinical research projects and case reports in interventional and structural cardiology. His current work emphasizes innovative catheter-based therapies for valvular heart disease and the integration of clinical examination with advanced imaging techniques. He is committed to advancing patient-centred cardiovascular care through research and education.

## Supplementary Material

ytag735_Supplementary_Data

## Data Availability

The data underlying this article are available from the corresponding author upon reasonable request and in accordance with the patient confidentiality regulations.
